# Supplementary figures and images for: Duplication and expression of Sox genes in spiders
Source: BMC Evol Biol. 2018 Dec 27;18:205. doi: 10.1186/s12862-018-1337-4 (PMC6307133; doi:10.1186/s12862-018-1337-4)

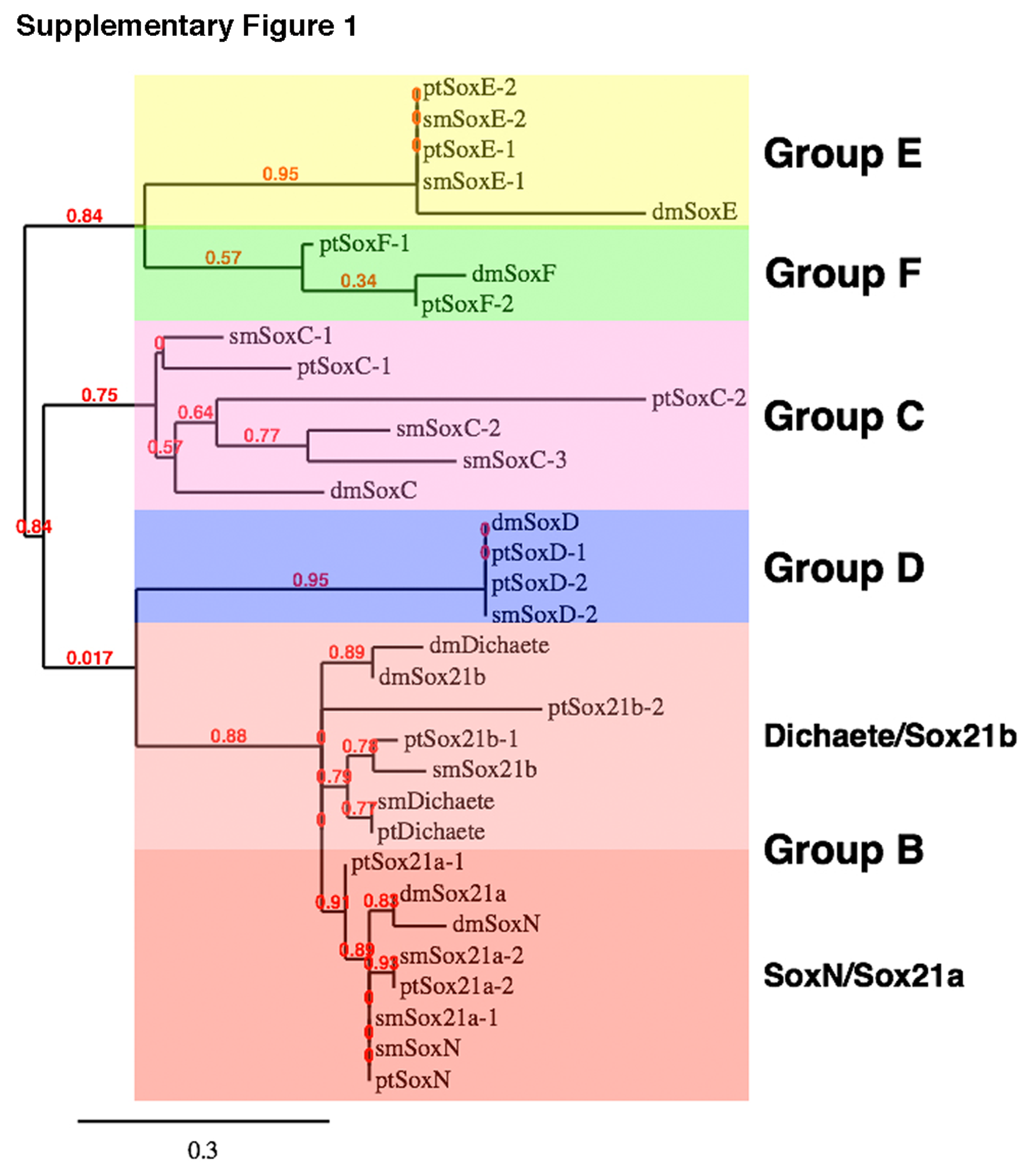

Supplement: Supplementary file 2 — Figure S1. Phylogeny of Group B Sox HMG domains PhyLM tree and multiple sequence alignment of group B HMG domains from Mus musculus (Mm), Drosophila melanogaster (Dm), Anopheles gambiae (Ag), Tribolium castaneum (Tc) Parasteatoda tepidariorum (Pt) and Stegodyphus mimosarum (Sm). Branch support values from PhyML are indicated in red. Arrow indicates the conserved Isoleucine reside indicative of invertebrate Dichaete/Sox21b class genes [15]. (PNG 849 kb) [file 12862_2018_1337_MOESM2_ESM.png]

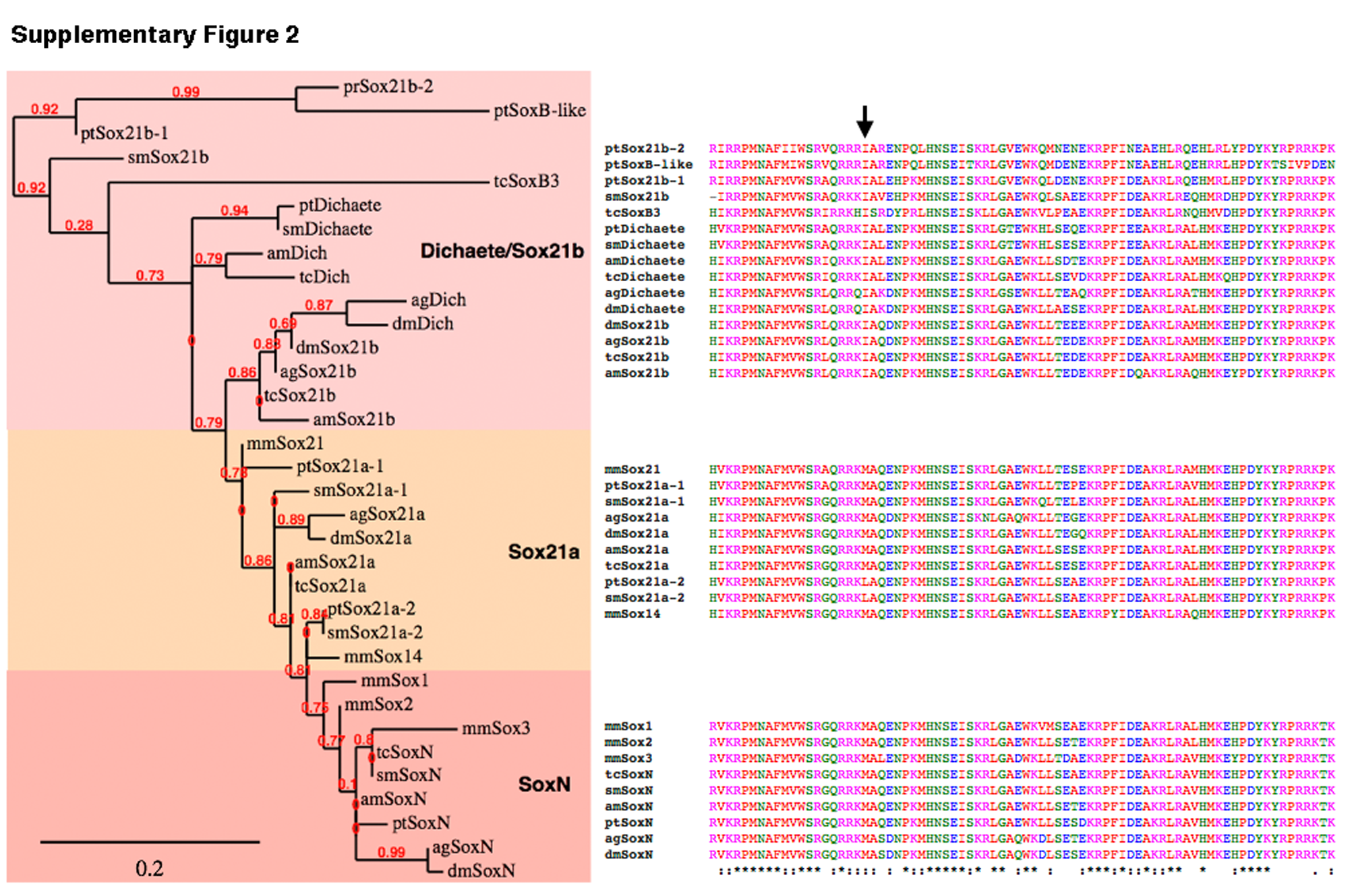

Supplement: Supplementary file 3 — Figure 2. Phylogeny of full-length Sox proteins from Drosophila and spiders. PhyLM tree of Sox genes from D. melanogaster (Dm), P. tepidariorum (Pt) and S. mimosarum (Sm) based on available full-length protein sequence (Additional file 1: Table S1). Branch support values from PhyML are indicated in red. (PNG 1624 kb) [file 12862_2018_1337_MOESM3_ESM.png]

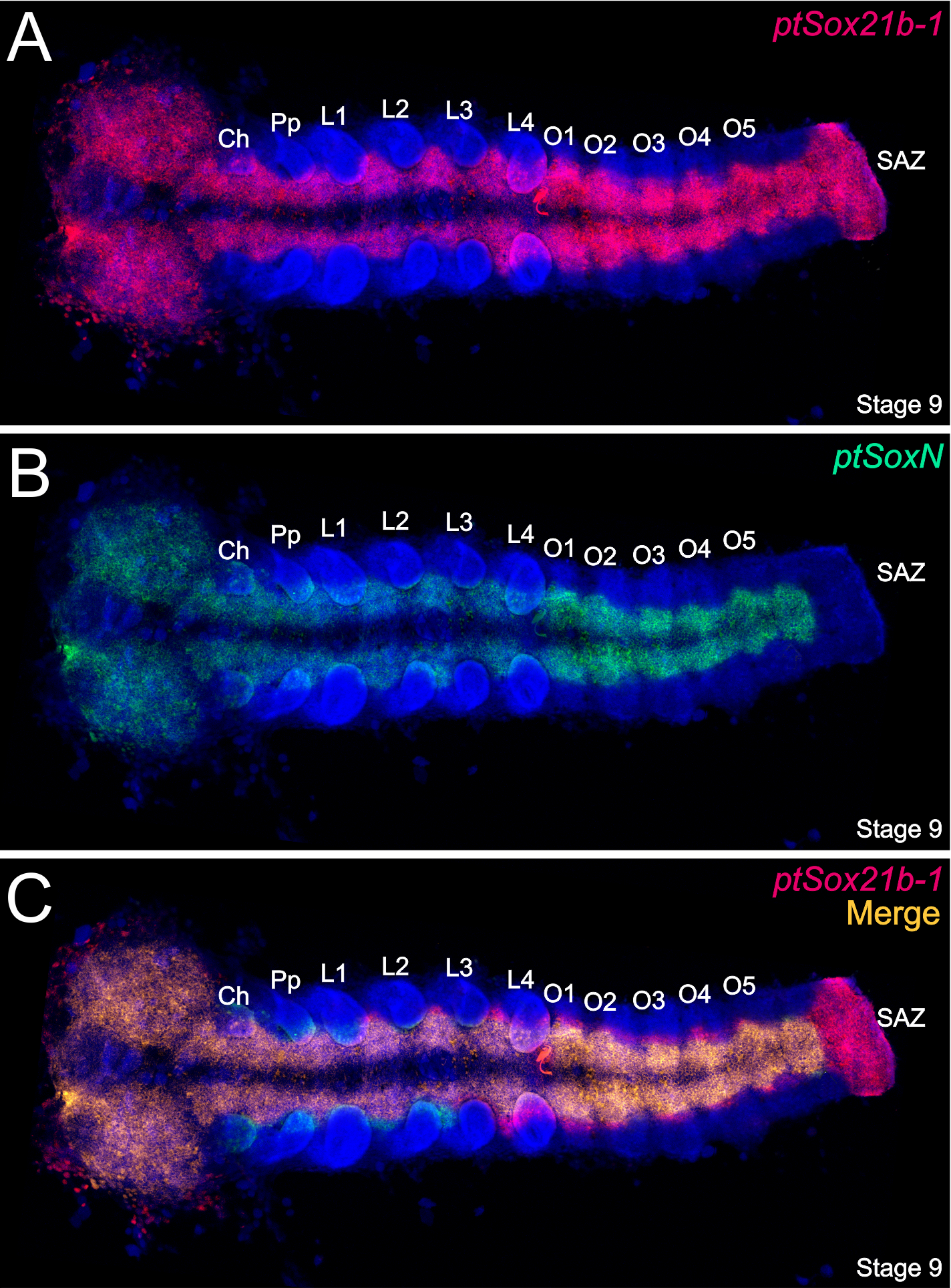

Supplement: Supplementary file 6 — Figure S3. Double Fluorescent in situ Hybridization Double in situ hybridization with (A) digoxigenin-labelled pt-sox21b-1 in red and (B) fluorescein pt-SoxN in green. C) Merged figures A and B shows the overlap. (PNG 2968 kb) [file 12862_2018_1337_MOESM6_ESM.png]
