# Supplementary material for: Duplication and expression of Sox genes in spiders
Source: BMC Evol Biol. 2018 Dec 27;18:205. doi: 10.1186/s12862-018-1337-4 (PMC6307133; doi:10.1186/s12862-018-1337-4)
Supplement: Supplementary file 7 — Table S4. Genes, primers sequences and sizes for all the fragments used for in situ hybridisations. (DOCX 15 kb) [file 12862_2018_1337_MOESM7_ESM.docx]

| **Gene** | **Primer Forward** | **Primer Reverse** | **Fragment Size** |
| --- | --- | --- | --- |
| Dichaete | 5’ GCGCTGCACATGAAAGAACA 3’ | 5’ CGGACTCTGTGGTACCG 3’ | 534bp |
| Sox 21-A | 5’ TGGCGATCACGTTAAGAGGC 3’ | 5’ GTGGGAAAGTACGTCGGAGG 3’ | 627bp |
| Sox 21-B | 5’ TCGGTACCTTACATACGGGTG 3’ | 5’ GATAGATGCTCGGTGGGGTG 3’ | 687bp |
| SoxNeuro | 5’ AAGAAAGACGCCGAACGAGT 3’ | 5’ AAGAAAGACGCCGAACGAGT 3’ | 500bp |
| Sox B-1 | 5’ AGGCCTTCAAATCAACGAGACT 3’ | 5’ AGCGCAGAAGTAACTGATGGA 3’ | 516bp |
| Sox B-3 | 5’ CGTCCCCGACGAAAACCTAA 3’ | 5’ CGGGGAACACTGATGTTGGA 3’ | 670bp |
| Sox C-1 | 5’ TGGGGGAGCTCTTCGACATA 3’ | 5’ GTGGACGGGGCTTTAGTCAA 3’ | 760bp |
| Sox C-2 | 5’ GCGTACAGCTGGGAAAGAGA 3’ | 5’ CTGAAGTCAGGCAACCGTCT 3’ | 591bp |
| Sox D-1 | 5’ ACCTGCAATTAATGAAGCGCC 3’ | 5’ AAAGCAGCAGGATGAGCTAC 3’ | 627bp |
| Sox D-2 | 5’ GGTTGTTACAGTGGGAGCGA 3’ | 5’ TTGCAGTGGTAGGTTGAGGC 3’ | 709bp |
| Sox E-1 | 5’ TATGGTTTGGGCTCAGGCAG 3’ | 5’ CACTGTTCGTCCATGAGGCT 3’ | 645bp |
| Sox E-2 | 5’ GCTGAGAGGCTAAGGTGCAA 3’ | 5’ CGTATTGGTGGTGAGGTGCT 3’ | 601bp |
| Sox F-1 | 5’ AAGCAGAACGTCTCCGACAG 3’ | 5’ TTGAAGCAGCAGAGAGAGCC 3’ | 675bp |
| Sox F-2 | 5’ TTTTCAGCAGGATCCACCCC 3’ | 5’ GCATATGCTGCACCCTCAATC 3’ | 666bp |

**Supplementary Table 2.** Genes, primers sequences and fragment sizes for all the transcripts.
